# Sexual dimorphism of the immune system predicts clinical outcomes in glioblastoma immunotherapy: A systematic review and meta-analysis

**DOI:** 10.1093/noajnl/vdac082

**Published:** 2022-05-27

**Authors:** Jack M Shireman, Simon Ammanuel, Jens C Eickhoff, Mahua Dey

**Affiliations:** Department of Neurosurgery, University of Wisconsin School of Medicine and Public Health, UW Carbone Cancer Center, Madison, Wisconsin, USA; Department of Neurosurgery, University of Wisconsin School of Medicine and Public Health, UW Carbone Cancer Center, Madison, Wisconsin, USA; Department of Biostatistics and Medical Informatics, University of Wisconsin School of Medicine and Public Health, Madison, Wisconsin, USA; Department of Neurosurgery, University of Wisconsin School of Medicine and Public Health, UW Carbone Cancer Center, Madison, Wisconsin, USA

**Keywords:** glioblastoma, glioma, immunotherapy, sexual dimorphism

## Abstract

**Background:**

Biological differences based on sex have been documented throughout the scientific literature. Glioblastoma (GBM), the most common primary malignant brain tumor in adults, has a male sex incidence bias, however, no clinical trial data examining differential effects of treatment between sexes currently exists.

**Method:**

We analyzed genomic data, as well as clinical trials, to delineate the effect of sex on the immune system and GBM outcome following immunotherapy.

**Results:**

We found that in general females possess enriched immunological signatures on gene set enrichment analysis, which also stratified patient survival when delineated by sex. Female GBM patients treated with immunotherapy had a statistically significant survival advantage at the 1-year compared to males (relative risk [RR] = 1.15; *P* = .0241). This effect was even more pronounced in vaccine-based immunotherapy (RR = 1.29; *P* = .0158).

**Conclusions:**

Our study shows a meaningful difference in the immunobiology between males and females that also influences the overall response to immunotherapy in the setting of GBM.

**Key Points**

- Female sex-linked genes are tied to immunological responses.
- Females have better outcomes during GBM immunotherapy treatments.

**Importance of the Study**

Our study adds to a growing body of literature examining sex differences in male and female immunology. We demonstrate both using large-scale omics data sets, as well as clinical trials, that female sexually dimorphic genes are tied to immunological responses, and that females have better outcomes during glioblastoma immunotherapy treatments. This data is critical to better inform treatment practices and further crystalizes the need for balanced trial design and prospective reporting of sex as a variable across all GBM clinical trials.

Sexual dimorphism, a well-described phenomenon in the animal kingdom, is increasingly being recognized as an important aspect of human health and disease pathophysiology.^1,2^ Emerging literature suggests that the sex differences in human physiology and pathophysiology exist across all stages of life, implying a more fundamental role of sex chromosomes than simply reproduction and hormone secretion. Sex differences in the rate of fetal, as well as placental growth, have now been recognized as early as the blastocyst stage,^3^ much earlier than adrenal and gonad development. Furthermore, the establishment of transcriptional differences between males and females both broadly, as well as immunologically, has been documented.^2,4,5^ Epidemiological studies have shown that sexual dimorphism plays a role in prevalence, severity, presentation, clinical course, and therapeutic outcomes of many common diseases such as cardiovascular disease, autoimmune disease, asthma, and cancer.^4^ However, this important variable is not commonly considered while studying various disease processes in basic science, translational work, and clinical trials, restricting the generalizability of study findings. Because of this, current available literature in almost all disease processes is highly skewed and biased toward males.^4,6,7^

Extensive genetic polymorphism is one of the most important hallmarks of the human immune system. For example, the human leukocyte antigen (HLA) loci are some of the most polymorphic in the entire genome.^8^ Sexual dimorphism in the biology of the immune response between sexes has been illuminated in both the innate and adaptive immune responses to infectious agents.^7,9,10^ There are differences between the sexes in the population of cells that make up the innate immune response with males tending to have a higher frequency of natural killer cells as well as innate lymphoid cells compared to females.^11^ Sex differences also influence production, secretion, and biological function of certain chemokines and cytokines, the critical regulators of immune response. In response to lipopolysaccharide (LPS) stimulation male peripheral blood mononuclear cells (PBMC’s) secrete more tumor necrosis factor (TNF) than female PBMC’s.^12^ Upon TLR9 activation male PBMC’s also release more interleukin 10 than their female counterparts.^13^ Specifically, neutrophils isolated from males either at basal levels, or when stimulated with LPS, secrete more TNF than female neutrophils.^12^ Finally, macrophages from males, when stimulated with LPS, secrete more CXCL10 than females.^14^ In females, phagocytic activity tends to be higher in both macrophages and neutrophils.^15^ Female PMBC’s also express more INF-a than male PMBC’s when stimulated with TLR7 ligands due to escape of X inactivation of the TLR7 gene.^16,17^ In fact, X inactivation is thought to play a large role in the overall dichotomous response of the male and female immune systems including the development of autoimmunity.^7,18,19^ (Table 1).

**Table 1.** Table Summarizing the Currently Published Differences Between Male and Female Immune Cell Subsets

Cell Type	Differential Effect in Females	Differential Effects in Males	Canonical Immunological Role
Dendritic cells	More efficient antigen presentation	Literature Inconclusive	Responsible for antigen processing and professional antigen presentation^9^
Macrophages	Higher phagocytic capacity	Increased CXCL10 secretion after LPS stimulation, Increased TNF secretion	Phagocytic cells responsible for removal of dead or dying cells or cellular debris^14,76^
Natural Killer Cells	Literature inconclusive	Higher number in circulation in males	Respond rapidly to foreign infections and initiate a cytotoxic response without the need for secondary activation^11^
Neutrophils	Higher Phagocytic activity	More TNF-alpha secretion in Basal or LPS stimulated condition	Rapidly acting phagocytes that respond to inflammation and can ingest opsin coated microbes^12^
CD4^+^ T cells	Higher Count Present, more TH1 lineage after infection, more INF-y on stimulation	Tend to produce more IL17 on stimulation	Critical in directing the responses of both B cells and Cytotoxic T cells through cytokine secretion and co-stimulation^20,21^
CD8^+^ T cells	Higher CD4/CD8 Ratio Present	Higher CD8 cell total frequency	Cytotoxic T cells recognize and kills infected or damaged cells in tissues via enzymatic degradation^20,21^
T-Regulatory Cells	Possibly less effective/robust in females (autoimmunity)	Higher frequency in males	Subset of T cells critical for maintaining tolerance to self-antigens and preventing autoimmune disease as well as downregulating effects of effector T cells^25^
B cells	Higher numbers present in circulation	Literature inconclusive	Participate in humoral immunity by generating antibodies to antigens encountered and bound to their B cell receptors^11,22^
Microglia	Possess a more developmental/repair like signature	Possess an inflammatory signature	The resident macrophages of the CNS they use phagocytosis to ingest damaged neurons, synapses, or other cellular debris in the CNS^29^
MDSC	Granulocytic MDSC enrichment in blood	Monocytic MSDC enrichment in tumor microenvironment	Immature myeloid cells which negatively impact the functions of T cells and Natural Killer cells^64,65^

Abbreviations: CNS, central nervous system; LPS, lipopolysaccharide; MDSC, myeloid-derived suppressor cell.

Similar differences between the male and female adaptive immune system have also been well characterized. Females tend to have a higher CD4^+^ T cell count and a higher CD4/CD8 T cell ratio when compared to age-matched male controls.^11,20^ Males tend to have a larger distribution of CD8^+^ T cells.^11^ Females were also shown to have a more active T cell repertoire after stimulation, with antiviral and pro-inflammatory genes being expressed as well as more circulating CD4^+^ and CD8^+^ T cells present in the blood when compared back to males.^21^ Furthermore, females show a greater antibody response, higher B cell numbers, and demonstrate more efficient antigen presentation than males.^11,22,23^ Overall, with regards to adaptive immunity, it is generally accepted that females show a more active and responsive immunophenotype whereas males are more likely to have an immunosuppressive phenotype, with research demonstrating males have an increase in the number of regulatory T cells (Tregs) compared to females.^24,25^

Clinically, the sexual dimorphism in immune system biology is also apparent with females being less likely to die from infectious diseases caused by bacteria, viruses, fungi, or parasites.^10^ Females with active HIV infection also show less viral RNA in the blood than males, and have higher circulating immunoglobulin G and immunoglobulin M levels.^10,26^ Females also demonstrate significantly stronger responses to viral vaccines as well as a decreased likelihood to die from malignant cancers.^9^ Unfortunately, this increased immunological protection comes with a dramatic increase in risk of autoimmune disorders like Multiple Sclerosis, Aplastic Anemia, and Coeliac Disease. It is estimated that 80% of autoimmune disorders occur in females, however, diseases like systemic lupus erythematosus have an incidence rate of up to 90% in females.^22,27,28^

Sex differences in immunobiology are also starting to become apparent in the unique immune microenvironment of the central nervous system (CNS). In a mouse model, Villa et al. demonstrated that a sex-specific genetic signature is encoded in microglia, and that this signature is not lost even if transplantation is performed on an opposite-sex mouse.^29^ These distinct signatures demonstrate that microglia isolated from male mice are more likely to respond in an inflammatory way, whereas female microglia showed a developmental/repair type signature.^29^ Astrocytes isolated from male pups also show a more robust anti-inflammatory response to stimulation with LPS than female counterparts.^30^ Finally, microglia in both GBM and neurofibromatosis type 1 (NF1) models have demonstrated sex differences, with JAM-A functioning as a pathogenic microglial suppressor but only in females in a GBM mouse model.^31^ In an NF1 mouse model, male microglia showed a selective decrease in purinergic controlled phagocytosis due to deficits in cyclic adenosine monophosphate regulation.^32^ Further work also demonstrates sex as a critical prognostic factor in optic glioma-associated visual decline, as well as neuronal dysfunction, in children with NF1.^33^ These studies emphasize both the importance of sexual dimorphism in the CNS immunology, and the uniqueness of CNS immune system compared to the peripheral immune system.

Glioblastoma (GBM) is the most common primary CNS malignancy in adults with an average occurrence rate of 3.19/100,000 individuals.^34^ De novo, grade IV primary GBM is known to have a gender bias with an incidence rate 1.6 times higher in males.^34,35^ On the other hand, secondary GBM (now reclassified to grade IV astrocytoma based on WHO 2021 revised criteria), a lower grade glial neoplasm that progresses to a GBM, has an incidence rate that is female biased with a male to female ratio of 0.65.^36^ In the clinical trial setting, median survival post GBM diagnosis is 20.9 months with the addition of tumor treating fields compared to 16 months using the standard of care.^37^ There is a gender difference in survival in the first year of disease progression (36.7% males surviving vs 32.8% females surviving), however, the effect is lost as the disease progresses further.^34^

One of the biggest limitations for developing effective treatment strategies for GBM is extensive inter and intra-tumor heterogeneity which necessitates the moving of the field towards personalized therapeutic approaches like immunotherapy. Although immunotherapy is well studied, there is a lack of clinical trial data explicitly comparing the outcomes of males and females across immunotherapy treatments. In context of other solid cancers, a meta-analysis of clinical trials using immune checkpoint programmed death ligand-1 (PD-1) and CTLA-4 blockers showed a survival benefit for males in CTLA-4 treatment, but no direct sex-specific survival benefits associated with PD1 inhibition.^38^ However, a similar meta-analysis found that both sexes improve from checkpoint inhibitors compared to conventional treatments and was unable to find a sex difference in overall or progression-free survival (PFS).^39^ These conflicting reports emphasize a nuanced role of sexual dimorphism in the overall outcome of immunotherapy that likely depends on tumor location and surrounding immunobiology. Females have also been shown to be at significantly higher risk of adverse events during immunotherapy, chemotherapy, and other targeted therapies, when data from Southwest Oncology Group cancer research network was retrospectively analyzed.^40^ Critically, however, the immunotherapy dataset did not contain any brain cancer cases which further highlights the need for sex-specific analysis of both brain tumor biology as well as clinical adverse events.^41^ To understand the role of sexual dimorphism on the immune system and GBM immunotherapy outcomes, we analyzed sex-specific large gene expression datasets and conducted a meta-analysis of immunotherapy clinical trials in GBM in which we could stratify the survival data by sex.

## Methods

### Sex-Associated Gene Database and TCGA Mining

**Figure 1.**
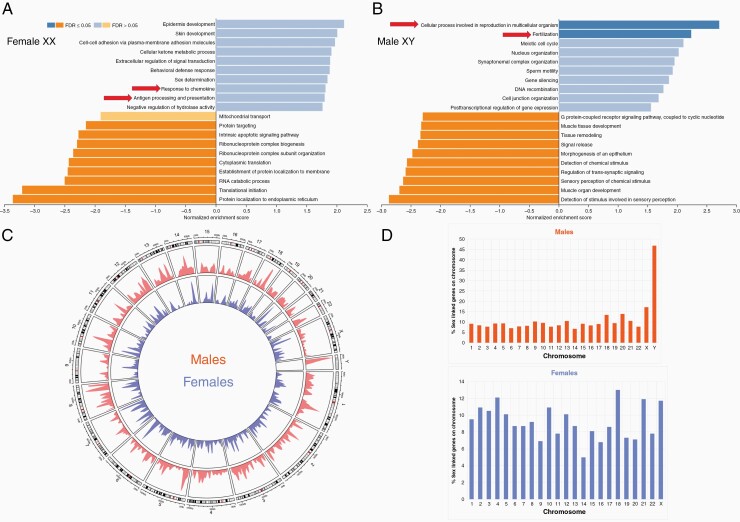
Gene set enrichment analysis of sexually dimorphic genes demonstrates enrichment for immunological pathways in females but not males. (A) GSEA conducted on female sexually dimorphic genes with greater than 2.5-fold upregulation. Arrows indicate immunological pathway upregulation for antigen processing and presentation (*P* < .008 false discovery rate (FDR) = 0.61) and chemokine response (*P* < .008 FDR = 0.67). (**B**) GSEA conducted on male sexually dimorphic genes with greater than 2.5-fold upregulation. Arrows indicate upregulation for cellular process in reproduction (*P* < .0001 FDR < 0.05) and fertilization (*P* < .0001 FDR < 0.05). (**C**) Whole chromosome landscape density plot of sexually dimorphic gene sets for males (outside of circle) in red and females (inside of circle) in blue. (D) Histogram showing the percent of sexually dimorphic genes that reside on each chromosome across both males and females.

**Figure 2.**
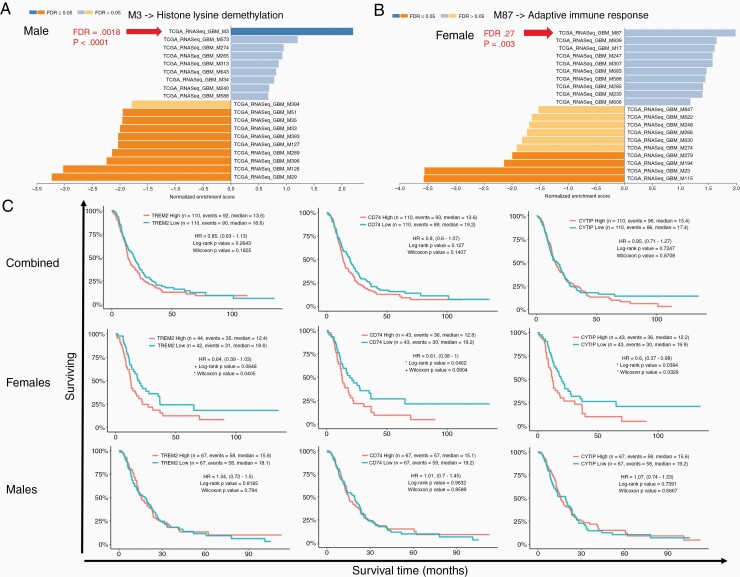
*Female* sexually dimorphic genes enrich for immunological pathways and impact survival in GBM. (A) GSEA conducted on male sexually dimorphic genes queried against the TCGA GBM RNA sequence modules which demonstrated enrichment for Module 3 Histone Lysine Demethylation (FDR = 0.0018 *P* < .0001). (B) GSEA conducted on female sexually dimorphic genes queried against the TCGA GBM RNA seq modules demonstrates enrichment for M87 Adaptive Immune Response (FDR = 0.27 *P* < .003). (C) Kaplan Meier survival curves from the CGGA dataset displaying survival time for TREM2 (left column), CD74 (center column), and CYTIP (right column). Survival is separated by sex with sexes combined (top) females only (middle) and males only (bottom). Both Log-rank and Wilcoxon *P*-values are reported. +*P* < .10; *P < .05.

The sex-associated gene database (SAGD)^42^ was utilized for data download while mining/manipulation of the data was done using R. Chromosomal locations were determined from the SAGD lists using the R package biomaRt.^43^ The chromosomal density calculation and plotting were done utilizing Circlize^44^ according to standard parameters. The Cancer Genome Atlas (TCGA) data was visualized using gliovis.^45^ Statistics on survival data were calculated by gliovis and reported using standard cutoffs. Both Wilcoxon and Log-Ranked statistical tests were performed on and reported for survival plots as is standard for gliovis. Log-Ranked tests give equal weights to all death events while Wilcoxon tests place more weights on earlier death events which can be somewhat misleading in some datasets. For male and female stratification of survival data the Chinese Glioma Genome Atlas (CGGA) dataset was preferentially used as it contains more female participants than the TCGA dataset. For gene set enrichment analysis (GSEA) analysis utilizing the Webgestalt platform analysis of both normal and TCGA data was done. Normal data were enriched for pathway enrichment utilizing Kyoto encyclopedia of genes and genomes functional database (Figure 1) and GBM data was enriched from TCGA (156 adult transcriptomic cases with data available) using network enrichment (Figure 2). CGGA data was not used for transcriptomics since it was not available through this portal.

### Clinical Trial Eligibility Criteria and Search Strategy

We conducted a literature search and meta-analysis according to the Preferred Reporting Items for Systematic Reviews and Meta-Analyses^46^ and Meta-Analyses of Observational Studies in Epidemiology^47^ guidelines (Supplementary Tables 1 and 2). Our search strategy followed the Population, Intervention, Comparison, Outcome, Study type question format: In either primary or recurrent GBM patients was there a difference between males and females in response to immunotherapy? (Supplementary Table 3) We examined outcomes of percent overall survival (OS) as well as PFS at 1 year following immunotherapy treatment. For the literature search, we queried the PubMed and Cochrane databases with search terms (GBM and radio chemotherapy and immunotherapy, malignant glioma and radio chemotherapy and immunotherapy, GBM and immunotherapy, GBM and virus, GBM and cellular vaccination, GBM and peptide vaccination, GBM and checkpoint inhibitors, GBM and PD-1, GBM and CTLA-4, GBM and cytokine therapy, GBM and antibody, GBM and CAR-T, GBM and immune stimulation, GBM and immune microenvironment, and GBM and dendritic cell [DC] vaccination). Terms were used in PubMed with the filters Clinical trials phase II, III, IV, and date range of January 1, 2005–February 1, 2022, and in Cochrane with the filters for trials and date range of January 1, 2005–February 1, 2022. The literature search was conducted independently by both J.M.S. and S.A. and verified to match, and no automated search tools were employed. Studies were then filtered based on our pre-defined eligibility criteria detailed in Table 2.

**Table 2.** Inclusion and Exclusion Criteria for the Meta-analysis

Inclusion Criteria	Exclusion Criteria
• All GBM clinical trials involving immunotherapy with or without chemotherapy and radiation between January 1, 2005 and February 1, 2022. • Published phase II, III, and IV trials and designated as clinical trials phase II, III, and IV (explicit) in the text. • Immunotherapies included: Virus, Vaccines (cells and peptides), Checkpoint inhibitors, Cytokine driven responses, CAR-T cells, Small Molecule Inhibitors of Immune Pathways, cytosine-phosphate-guanine stimulation. • Trials in the setting of both primary and recurrent disease with no other neurological diseases • Trials include adults only (18+) • Trials published only in English (regardless of location conducted) • Trials must include data separated by sex for overall survival and/or progression-free survival • Trials limited to human subjects only	• Trials do not include data separated by sex. • Other malignant tumor present in trial population different than primary or recurrent GBM. • Presence of other neurological diseases in trial population. • Trials including children (under 18 years of age). • Trials published in a language other than English. • Trials not limited to human subjects. • Trials that are unpublished or not explicitly defined as Phase II, III, or IV in the text.

Abbreviation: GBM, glioblastoma.

The full search results are illustrated in Supplementary Figure 1. Briefly, after the above filters were applied to the search results from each database, 710 studies remained and were screened based on titles and abstracts to determine if the study met our inclusion/exclusion criteria (Table 2). Two hundred and six studies remained after this screening step which were screened by full manuscript screening to accurately assess for inclusion/exclusion criteria, specifically the inclusion of the sex of the participants in the study. In total 10 studies met our full inclusion criteria while a further 48 met all inclusion criteria except for inclusion of the sex of participants in the study. No missing data or summary statistics were present in our selected trials, and data from trials was visualized in grouped fashion using statistical software package meta rather than individually.

### Data Collection

All data were extracted from the main manuscript, figures, and Supplemental Material within the published trials. Percent OS at 1 year and PFS at 1 year were both calculated within R along with other meta-analysis statistics such as relative risk (RR), heterogeneity score (I^2^), and 95% CIs (methods below). Data extraction was done by two independent investigators (J.M.S. and S.A.) and extracted data was confirmed by senior investigator (M.D.).

### Statistical Analysis

Statistical analyses were conducted using R Studio, Version 1.4.1103. The meta-analyses, tests for heterogeneity, and publication bias analysis was performed using the R package meta version 4.15.^48^ OS and PFS at 1 year were collected and stratified according to the sex of participants reported by the trials. In our analysis females were considered the experimental group while males the control group. The metabin function was used to calculate RR between the groups using the Mantel-Hanzel method. A random effects model was used to estimate the overall effect and fit a prediction interval. The DerSimonian-Laird estimator was used to estimate between study variances. Heterogeneity between our studies was also assessed with the I^2^ statistic and the Cochran Q-Test that is derived from the chi-squared distribution. An I^2^ statistic > 50% and the *P* value of the Q-Test < 0.10 indicated the presence of heterogeneity. Publication bias was also assessed in our analyses using inspection of contoured funnel plots.

### Quality Evaluation of Clinical Trials

Trials used for analysis in this study^49–58^ were methodologically evaluated according to the revised Cochrane risk-of-bias tool for randomized trials.^59^ This system categorizes the studies as low, unclear, or high risk of bias, according to the following parameters: random sequence generation, allocation concealment, blinding of participants and personnel, blinding of outcome assessment, incomplete outcome data, selective reporting, and other bias. Most of our trials scored in the high-risk category for bias except for 1 trial (Cho) which scored as some concerns for bias (Supplementary Figure 2). This high bias score was driven by the lack of randomization and blinding in nearly all the trials studied. In every trial studied it was known to patients and caregivers that they were in an intervention group due to the invasive nature of the administered treatments and/or lack of a control arm. Furthermore, in our trial population follow up care if progression did occur on trial was either not discussed (Cho, Geletneky, Izumoto, Lim, Vik-Mo, Schalper, Wheeler) or was left up to individual physician discretion (Inoges, Pellegatta, Sampson) leading to discrepancy in follow up treatments. Our included trials in general did not suffer from data loss (Vik-Mo did lose 4/11 total patients) or selective reporting of results.

We also evaluated the quality of our included studies, as well as the statistical outcomes we found, using the Grading of Recommendations, Assessment, Development, and Evaluation system (GRADE).^60^ The GRADE system considers trial design, risk of recruitment/randomization bias, inconsistency, indirectness, and imprecision of results to produce a final score of confidence in the effect found during an analysis which ranges from High to Very Low. Under the GRADE criteria, each of our discovered effects scored in the very low confidence grade (Supplementary Table 4). This is due to serious bias in the lack of randomization in all trials examined except 1 (Cho), the indirectness present in many of the studies including differences in population studied (primary or recurrent) or immunotherapy modality used (see Table 3), as well as the overall small sample sizes in each study. In general, the low quality of the trials that we were able to analyze in this study due to restrictions on data availability should inspire caution when interpreting the effects found, as the small sample sizes, lack of randomization/blinding, confounds of treatments, and mix of primary and recurrent tumors may lead to either over or underestimations of the true effect.

**Table 3.** Participant Characteristics From All Analyzed Studies

First Author	Year of Publication	Trial Phase	Country of Publication	Age	Patients Included (n)	Male (n)	Female (n)	Trial Conducted on Primary or Recurrent GBM	Immunotherapy Type Used	Route of Delivery	Median Overall Survival Immunotherapy Group (Months)
Cho	2012	II	China	32–68	18	8	10	Primary	Tumor Cell Lysate Dendritic Cell Vaccine	Intravenous	31.9
Sampson	2010	II	USA	29–67	18	13	5	Primary	Epidermal growth factor receptor(VIII) Specific Tumor Vaccine	Intradermal	26
Geletneky	2017	I/II	Germany	42–76	18	14	4	Recurrent	ParvOryx Oncolytic Parvovirus	Intratumoral and Intravenous	15.2
Izumoto	2008	II	Japan	20–76	21	14	7	Recurrent	9-mer WT1 Peptide Vaccine	Intradermal	10.5
Lim	2021	I/II	South Korea	27–69	14	6	8	Recurrent	Adoptive Immune Cell Therapy (ex-vivo expanded and activated NK and T cells from patients)	Intravenous	22.5
Osland Vik-Mo	2013	I/II	Norway	46–63	7	4	3	Primary	Dendritic Cell Vaccination against patient derived cancer stem cell	Intravenous	8.9
Schalper	2019	II	USA	32–73	30	20	10	Both	Nivolumab	Intravenous	7.3
Wheeler	2008	II	USA	22–74	34	24	10	Both	Tumor Cell Lysate Dendritic Cell Vaccine	Intravenous	Not reported for overall population
Inoges	2017	II	Spain	42–70	29	14	15	Primary	Tumor Cell Lysate Dendritic Cell Vaccine	Intravenous	23.4
Pellegatta	2018	II	Italy	23–70	24	16	8	Primary	Tumor Cell Lysate Dendritic Cell Vaccine	Intravenous	20.1
Total					213	133	80				

Abbreviation: GBM, glioblastoma.

## Results

### *GSEA* for Sex-Specific Genes Demonstrates Enrichment for Immunological Pathways in Females But Not Males

To understand the role of sex-specific genes on overall immunological function in humans, we utilized the SAGD which curates a publicly available database of genes that have expression linked to sex differences across multiple species. For our purposes we were interested in studying the role of genes specifically upregulated in human males (XY) or females (XX) and whether they played a role in broad immunological pathways. From SAGD we obtained a list of genes with log2 expression values with (+) value indicating higher expression in males and (–) value indicating higher expression in females. The list was then filtered to include log_2_ values above 2.5 and with corresponding *P* < .01 and was separated by sexes, which resulted in gene sets of about ~3500 genes for females and ~7000 genes for males (Supplementary Table 5). We term these genes “sexually dimorphic” throughout the manuscript owing to their differential expression in males and females. These gene sets were then subject to GSEA using the web gestalt platform querying parameters for gene ontology and biological processes both across normal tissues (Figure 1) as well as the TCGA GBM dataset (Figure 2).^61^ We observed broad enrichment across both sexes of many responses that would be expected with sex-specific genes such as sex determination, nucleus organization, sperm motility, and DNA recombination (Figure 1A and B). However, in females we observed immune-related enrichment such as antigen processing and presentation (*P* < .008 FDR = 0.61) and chemokine response (*P* < .008 FDR = 0.67) (Figure 1A). The corresponding genes that were responsible for the enrichment seen in the female GSEA were response to chemokines: CXCL family (CXCL1,2,3,4,8) CCL13 and 26, CXCR4, and CCL13. Antigen processing and presentation: HLA-(DQB2,F,DRB5,DRA), TREM2, CD36, and CD74. This was absent in males where the most robust pathway enrichment was cellular pathways involved in reproduction (*P* < .0001 FDR < 0.05) and fertilization (*P* < .0001 FDR < 0.05) (Figure 1B). If parameters were expanded it was possible to pick up more immune-related enrichment, however, for most meaningful analysis we chose to limit the enrichment from GSEA to the top 10 terms.

We next wanted to determine if there was a chromosomal bias in sex-specific gene expression to understand if genes located on sex chromosomes alone accounted for most of the differences in sex-specific expression. A density plot of the sex-specific gene lists demonstrates that sex-related genes from both males and females are distributed across all chromosomes (Figure 1C). After removing duplicated genes from SAGD lists and filtering for protein coding genes only, percent of chromosomes occupied by sexually dimorphic genes were plotted by dividing the number of sexually dimorphic genes found from screening by the total number of protein coding genes present on the chromosome (Figure 1D). The male Y chromosome showed the highest concentration of sexually dimorphic genes while females, in contrast, show a much wider distribution of their sexually dimorphic genes with the frequency of genes occurring on the X chromosome like other nonsex chromosomes (Figure 1D.) These data suggest that sex-specific gene expression in females is not driven solely by the X chromosome, and this genome-wide distribution may be responsible for the overall robust immune response seen in females.

### Female Sex-Specific Genes Enrich for Immunological Pathways and Impact Survival in GBM

To understand if any sex-specific genes demonstrated pathway enrichment in the GBM setting, we utilized the sex-specific genes gathered from SAGD examined in Figure 1 and preformed exploratory GSEA on the TCGA GBM RNAseq dataset using the web gestalt portal. We found a gender bias in that the male gene sets enriched for modules having to do with transcription or histone modifications, such as Module 3 Histone Lysine Demethylation (*P* < .0001 FDR = 0.0018), whereas the female gene sets showed enrichment for adaptive immune response module 87 (*P* < .003 FDR = 0.27) (Figure 2A and B). We sought to understand if the genes at play in female specific enrichment impacted overall patient survival, and obtained this data using CGGA dataset, which can be subset by sex within the Gliovis portal. Of the six genes (HLA-DMA, IL18, HLA-DRA, TREM2, CD74, CYTIP) from female set that were enriched in the adaptive immune response model, three (TREM2, CD74, and CYTIP) showed a statistically significant OS benefit in the female patient population only. Analysis of Trem2 survival in females between high and low expression demonstrated a difference in median survival of 6.2 months (hazard ratio [HR] 0.64, Log-rank *P* value = .0648, Wilcoxon *P* value = .0405). CD74 showed a difference in median survival of 6.4 months (HR 0.61, Log-rank *P* value = .0462, Wilcoxon *P* value = .0904). CYTIP showed a median survival difference of 7.7 months (HR 0.60, Log-rank *P* value = .0394, Wilcoxon *P* value = .0329) (Figure 2C). There were other immune specific gene sets such as, module 17 Cytokine Production and Receptor Activity (*P* < .021, FDR = 0.54) however, due to the low FDR score we believe it is of less function consequence. Our results indicate that female sex-associated genes can enrich for an immunological response in GBM tumors and do provide some survival benefit. These data also demonstrate the critical need for the ability to separate out survival by sex as these effects are masked if sex is not considered as an independent variable.

### Clinical Trial Outcome Data/Meta-Analysis

Since our analysis of omics datasets validated the differential effect of sex-specific immune genes on the overall outcome of GBM patients, we hypothesized that there might be a difference in the overall outcome of immunotherapy in GBM patients based on sex. To test this hypothesis, we performed a meta-analysis of all available immunotherapy clinical trials for primary and recurrent GBM based on pre-defined inclusion criteria (Table 2). Our literature search ultimately yielded 10 publications that met our inclusion criteria with a total of 213 patients able to be analyzed (133 males and 80 females). The participants in the trials ranged in age from 20 to 76 while the majority demographic was Caucasian. The trials were conducted in the USA, Germany, Japan, South Korea, Norway, Spain, Italy, and China. 5 of the 10 trials were conducted on exclusively primary GBM, while 3 were conducted on exclusively recurrent GBM, and 2 had mixed populations (Summarized in Table 3).

### Females Display Statistically Significant Increase in Survival at 1-year Post-Immunotherapy Treatment Compared to Males

**Figure 3.**
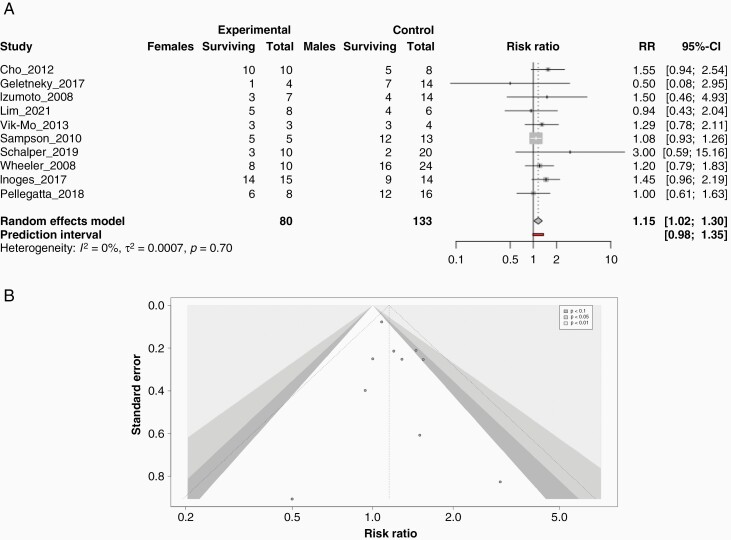
Females display statistically significant increase in survival at 1-year post-immunotherapy treatment compared to males. (A) Forrest plot for the trials analyzing patient overall survival at 1-year post-diagnosis stratified by sex. The plots report relative risk and 95% CIs computed using a random effects model as well as the I^2^ score for statistical heterogeneity. (B) Contour funnel plot assaying publication bias among the studies included in the model used for analysis.

We first set out to examine OS at 1 year across males and females who had received any type of included immunotherapy to determine if there was a baseline sex advantage present. Analysis of the clinical trial data (80 females, 133 males) from the 10 trials included in this study showed a significant increase in the number of females surviving at 1 year after an immunotherapy treatment when compared to males (RR = 1.15; 95% CI, [1.02 to 1.30] ; *P* = .0241; I^2^ = 0%) (Figure 3). We also examined publication bias among our studies using a contoured funnel plot and observed slight asymmetry from standard indicating the presence of publication bias within our analysis (Figure 3B).

### No Statistically Significant Difference in PFS at 1 year Between Sexes

Next, we analyzed if this OS advantage is seen in females translated to better PFS at 1 year as well. For this analysis, our trial data set contained 8 studies that allowed us to analyze PFS at 1 year in a total of 55 females and 95 males. There was no significant difference in the PFS between the sexes observed (RR = 1.37’ 95% CI, [0.98 to 1.91]; *P* = .0673; I^2^ = 0%) (Supplementary Figure 3). Again, we examined publication bias using a contoured funnel plot and observed a slight shift from standard likely due to the small sample size and large magnitude of the standard errors of effects for the studies included (Supplementary Figure 3).

### Females Display a More Robust Survival Advantage at 1-year Post-DC Immunotherapy Treatment Compared to Males

**Figure 4.**
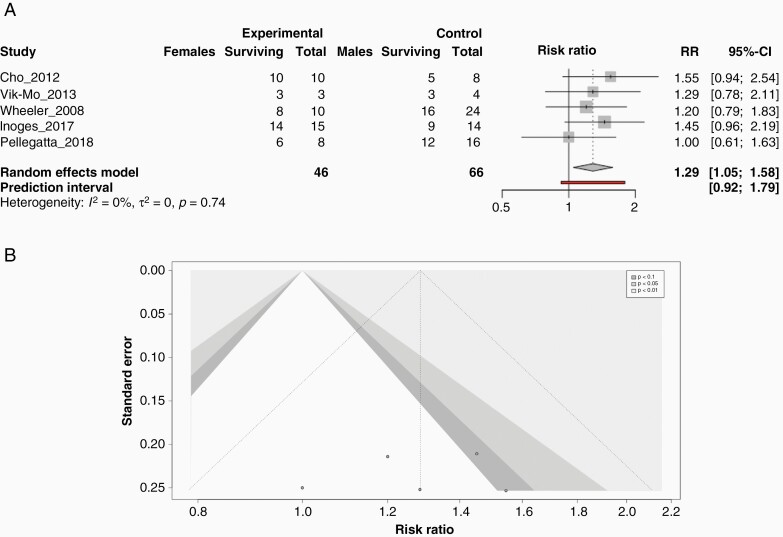
Females display a more robust increase in survival at 1-year post-dendritic cell immunotherapy treatment compared to males*. (*A) Forrest plot for dendritic cell vaccine specific trials analyzing overall survival at 1-year post-diagnosis stratified by sex. The plots report relative risk and 95% CIs computed using a random effects model as well as the I^2^ score for statistical heterogeneity. (B) Contour funnel plot assaying publication bias among the studies included in the model used for analysis.

Finally, due to the myriad of basic biological research suggesting females contain a more robust antigen presentation capacity, we believed it would be prudent to separately check the effects of an immunotherapy that could directly leverage this biological advantage. We examined from our population of trials only the ones that utilized autologous dendritic cell (DC) therapy where the patient’s own immune cells are primed to the tumor cell lysate to encourage more robust antigen presentation. We were able to examine 46 females and 66 males and observed a strong statistically significant survival benefit for females at 1 year when compared to males (RR = 1.29; 95% CI, [1.05 to 1.58]; *P* = .0158; I^2^ = 0%) (Figure 4A). This effect was larger than our combined immunotherapy OS effect hinting that immunotherapy targeted to a sex-specific strength in the female immune system may provide enhanced benefit. We also examined publication bias in this trial set using a contour funnel plot and observed a significant shift from normal likely due to the overall small number of studies we were able to gather data from causing a significant publication bias (Figure 4B).

## Discussion

These data demonstrate sexually dimorphic genes tend to enrich for immunological signatures in females but not males. Furthermore, females have a much wider chromosomal distribution of their sexually dimorphic genes compared to males, in which many sexually dimorphic genes reside on the Y chromosome. These genes can play a role in stratifying survival outcomes in a large GBM patient dataset, but only if the sexes are separated out and analyzed individually. We then examined if any of the current immunotherapy trials conducted in GBM showed a sex bias in terms of OS or PFS at 1 year and found a significant increase in survival in females who had undergone immunotherapy when compared to males. Furthermore, when we looked specifically at DC-based immunotherapy, we found a larger increase in survival at 1 year compared to males. Even though the effect size is small, we should keep in mind that is based on a small study cohort, it is worthwhile to re-analyze the data stratified by sex from the larger trials to assess the effect size to analyze the effect of sexual dimorphism on the outcome of a disease that is in desperate need for effective therapy. Finally, our analysis of PFS between the sexes did not demonstrate a statistically significant difference. Although we were able to find a sex-specific effect, there was a significant amount of methodological and publication bias observed in our clinical trial set as indicated by our funnel plots and GRADE analyses, which needs to be considered when interpreting these findings. Our meta-analysis was severely limited by the inability to gather large portions of relevant data to answer our proposed question, as it is not standard practice to include sex as an analyzable factor in the reporting of clinical trials. To truly understand the overall therapeutic potential of immunotherapy, it is critical to update our trial design and reporting to incorporate upfront integration of sex as a variable.

Emerging literature aimed at understanding the pathophysiology of GBM convincingly shows a clear sex difference in the immune biology between males and females. In a preclinical study, Sun et al. showed that sex-specific retinoblastoma protein inactivation drives the prevalence of the mesenchymal subtype of GBM in males.^62^ Additional studies by the Rubin group have led to the discovery that males and females respond to the standard of care for GBM differently, as well as harbor different gene expression profiles within their tumors, suggesting that a one size fits all targeting strategy may be futile.^63^ Myeloid-derived suppressor cell (MDSC) subsets have also been examined as important regulators of the immune environment, with monocytic MSDCs playing a role in supporting primary tumor dissemination through an epithelial to mesenchymal transition and promotion of cancer stem cell growth, while granulocytic MDSC’s reverse this transition and support primary tumor growth.^64^ Bayik et al. have shown a sex-specific phenotype in MDSCs where male mice with GBM tumors had monocytic MDSCs localized to the tumors, whereas female mice had enrichment of granulocytic MDSCs in their blood.^65^

Clinically, sex-specific patterns of tumor growth, measured via imaging, have also been identified by Whitmire et al.^66^ They demonstrate that tumor cell diffuse invasion rate is negatively correlated with survival in females but not males, while the size of the tumor overall is correlated with survival in males but not in females. Furthermore, male incidence bias has been seen clinically in both high- and low-grade brain tumors in data from six different countries with tumors such as astrocytomas, oligodendrogliomas, ependymoma, and medulloblastoma.^67^ These studies suggest a meaningful biological difference between the sexes when it comes to the progression and treatment of GBM and brain cancer in general, however, the clinical data needed to accurately answer this question are often prohibitively hard to access at present. In fact, our study is the first to be able to examine these possible sex-specific effects with actual clinical trial data in GBM immunotherapy and begin to understand whether these predicted biological differences seen in basic science research translate to outcome differences in clinical practice.

Our meta-analysis also highlights a critical point that needs to be specifically discussed in the current clinical literature to more the field of GBM immunotherapy forward. Our OS analysis at 1 year contained trials that utilized a wide variety of immunotherapies that target unique aspects of the immune system. This certainly played a role in the underlying variation in the Risk Ratios between the studies, as the biology between the male and female immune systems is highly varied.

One well-established immunological benefit that females possess is a more robust antigen presentation response driven by DCs. In fact, antigen presentation and cytokine secretion were the two top female immune-related hits in our GSEA analyses utilizing our sexually dimorphic genes in both normal individuals as well as TCGA data, signaling a likely functional outcome for these specific sets of genes is possibly related to adaptive immunity and the priming of the immune response in both normal and tumor conditions. As we know from previous research that females have more robust DC subsets, specifically type 1 DCs which play a role in antitumor immunity by priming cytotoxic T cells.^9^ Thus, it is not surprising that there could be a clinical benefit for DC vaccinations, which prime DCs for antigen presentation using the patient’s own apoptotic tumors cells,^68^ specifically in females, even if they show limited efficacy in the male population. We were able to examine this directly and show a more robust survival benefit in females compared to males in our clinical population. These results suggest that the better understanding of the fundamental differences between male and female immune systems could lead to immunotherapies that specifically leverage those advantages to maximize treatment effectiveness. For example, males have been shown to have a higher overall number of CD8^+^ T cells, but also more Tregs, so immunotherapy targeting the formation of Tregs may have a differentially more beneficial effect in males where the starting population is higher than in females. Critically, these effects are likely to be masked or underestimated if sex is not properly reported and considered during the trial design. It’s also important to consider that a full understanding of male and female immunobiology in the complex microenvironment of brain is still in its infancy and may run counter to our current systemic understanding of the immune system.^29,30^

Of the trials included in our study that were not DC vaccination based, we had varying other modalities of immunotherapy including an oncolytic parvovirus, an EGFRVIII specific tumor vaccine, a Wilms tumor protein-1 specific vaccine, adoptive immune cell transfer therapy, and the anti-PD1 antibody Nivolumab. In this cohort only, the oncolytic virus showed a more robust effect in males than females (50% males surviving at 1 year vs 25% females) which is counter to what we would believe based on basic science research with females typically mounting the more robust viral antigen responses. However, it should be noted that the population of males in the study was much higher than females and could have introduced substantial bias (14 to 4). Unfortunately, due to lack of studies with sex data stratification, we were also unable to gather multiples of these types of studies as we could with DC vaccines, limiting our abilities to effectively draw more robust conclusions.

### Strengths and Limitations

A major limitation of our analysis is the lack of publicly available data on sex as a variable. Most clinical trials that we analyzed did not report outcomes stratified by sex in a way that allowed for effective analysis. In fact, 48 total studies needed to be excluded from our analysis solely for the fact that they did not include sex as an analyzable variable. Furthermore, some of the largest studies to date on immunotherapy in GBM^69–74^ fail to report or include in an accessible way their patient sex data limiting our analysis to smaller trials. If this were not the case, it would have provided more power to draw robust statistical conclusions as well as the freedom to analyze more modalities of immunotherapy independently. It is also certainly a limitation that our trials used several separate types of immunotherapies, further increasing the heterogeneity in our analysis. Each of these limitations likely were large contributors to the methodological and publication biases found during our assessment of both GRADE and risk of bias scores. All these factors raise the possibility that we are not capturing the true effect size, either in direction or magnitude. However, there is a known female survival advantage present in GBM regardless of treatment,^75^ which emphasizes differential role of the sex on overall outcome of this disease and raises the possibility that the implication of sexual dimorphism might extend beyond immunotherapy to all additional treatment modalities. Critically, it is impossible to accurately conclude any of this unless the current data limitations are resolved, and a comprehensive analysis is conducted based on sex for every clinical trial prospectively.

## Conclusion

In summary, we demonstrated that immune-associated sexual dimorphic genes show a bias in enrichment towards immunological stimulation only in females, that females show a wider chromosomal distribution of these sexually dimorphic genes, and that these genes can be correlated with better survival in large GBM patient datasets. We are also able to demonstrate that these survival effects are only seen when the populations are analyzed separately and are masked if the data are analyzed as a whole, highlighting the fact that these differences while meaningful are subtle and need to be rigorously tracked. Clinically, we were able to demonstrate a significant difference in OS at 1-year post-immunotherapy treatment, which was further increased when we analyzed DC-Vax trials specifically. Findings from our study convincingly show that to accurately embrace personalized therapies for cancers such as GBM, it is of paramount importance to update our clinical study design and recruitment practices to include sex as an independent diagnostic as well as prognostic variable, and prospectively report findings stratified by sex.

## Supplementary Material

vdac082_suppl_Supplementary_Material

vdac082_suppl_Supplementary_Table_S5

## Data Availability

Data extracted from studies and used for analysis along with analysis code will be made available upon publication according to journal requirements. This analysis was not preregistered.
